# Spread of artemisinin-resistant *Plasmodium falciparum* in Myanmar: a cross-sectional survey of the K13 molecular marker

**DOI:** 10.1016/S1473-3099(15)70032-0

**Published:** 2015-04

**Authors:** Kyaw M Tun, Mallika Imwong, Khin M Lwin, Aye A Win, Tin M Hlaing, Thaung Hlaing, Khin Lin, Myat P Kyaw, Katherine Plewes, M Abul Faiz, Mehul Dhorda, Phaik Yeong Cheah, Sasithon Pukrittayakamee, Elizabeth A Ashley, Tim J C Anderson, Shalini Nair, Marina McDew-White, Jennifer A Flegg, Eric P M Grist, Philippe Guerin, Richard J Maude, Frank Smithuis, Arjen M Dondorp, Nicholas P J Day, François Nosten, Nicholas J White, Charles J Woodrow

**Affiliations:** a Myanmar Oxford Clinical Research Unit, Yangon, Myanmar; b Defence Services Medical Research Centre, Naypyitaw, Myanmar; c Department of Molecular Tropical Medicine and Genetics, Mahidol University, Bangkok, Thailand; d Mahidol Oxford Tropical Medicine Research Unit, Mahidol University, Bangkok, Thailand; e Faculty of Tropical Medicine, Mahidol University, Bangkok, Thailand; f Shoklo Malaria Research Unit, Mae Sot, Thailand; g Institute of Medicine 1, Yangon, Myanmar; h Department of Health, Ministry of Health, Naypyitaw, Myanmar; i Department of Medical Research, Upper Myanmar, Myanmar; j Department of Medical Research, Lower Myanmar, Myanmar; k WorldWide Antimalarial Resistance Network, Oxford, UK; l Centre for Tropical Medicine and Global Health, Nuffield Department of Medicine, University of Oxford, Oxford, UK; m Dev Care Foundation, Dhaka, Bangladesh; n Department of Genetics, Texas Biomedical Research Institute, San Antonio, TX, USA; o School of Mathematical Sciences, Monash University, Melbourne, Australia; p Medical Action Myanmar, Yangon, Myanmar

## Abstract

**Background:**

Emergence of artemisinin resistance in southeast Asia poses a serious threat to the global control of *Plasmodium falciparum* malaria. Discovery of the K13 marker has transformed approaches to the monitoring of artemisinin resistance, allowing introduction of molecular surveillance in remote areas through analysis of DNA. We aimed to assess the spread of artemisinin-resistant *P falciparum* in Myanmar by determining the relative prevalence of *P falciparum* parasites carrying K13-propeller mutations.

**Methods:**

We did this cross-sectional survey at malaria treatment centres at 55 sites in ten administrative regions in Myanmar, and in relevant border regions in Thailand and Bangladesh, between January, 2013, and September, 2014. K13 sequences from *P falciparum* infections were obtained mainly by passive case detection. We entered data into two geostatistical models to produce predictive maps of the estimated prevalence of mutations of the K13 propeller region across Myanmar.

**Findings:**

Overall, 371 (39%) of 940 samples carried a K13-propeller mutation. We recorded 26 different mutations, including nine mutations not described previously in southeast Asia. In seven (70%) of the ten administrative regions of Myanmar, the combined K13-mutation prevalence was more than 20%. Geospatial mapping showed that the overall prevalence of K13 mutations exceeded 10% in much of the east and north of the country. In Homalin, Sagaing Region, 25 km from the Indian border, 21 (47%) of 45 parasite samples carried K13-propeller mutations.

**Interpretation:**

Artemisinin resistance extends across much of Myanmar. We recorded *P falciparum* parasites carrying K13-propeller mutations at high prevalence next to the northwestern border with India. Appropriate therapeutic regimens should be tested urgently and implemented comprehensively if spread of artemisinin resistance to other regions is to be avoided.

**Funding:**

Wellcome Trust–Mahidol University–Oxford Tropical Medicine Research Programme and the Bill & Melinda Gates Foundation.

## Introduction

Artemisinin-based combination treatments are the mainstay of treatment for *Plasmodium falciparum* malaria globally, but artemisinin resistance, evidenced by delayed parasite clearance after artemisinin treatment, is now prevalent across an expanding area of southeast Asia.^1^, ^2^, ^3^, ^4^, ^5^, ^6^, ^7^ Artemisinin resistance is characterised by reduced susceptibility of the ring stage of parasite development^8^, ^9^ and is clearly associated with increasing rates of failure of artemisinin-based combination treatments in Cambodia^10^, ^11^, ^12^, ^13^ and Thailand.^14^

Mutations that change the primary aminoacid sequence of the so-called propeller region of the kelch motif-containing gene, known as K13, have been identified as a key causal determinant of artemisinin resistance in southeast Asia,^4^, ^15^, ^16^ acting through upregulation of unfolded protein response pathways.^17^ Various K13-propeller mutations have been documented in population surveys in the region, and when phenotypes are available in numbers sufficient enough to achieve statistical power, the most prevalent mutations are associated with delayed parasite clearance after artemisinin treatment^4^, ^5^, ^7^, ^18^ and reduced in-vitro responses.^19^, ^20^ No frequently occurring propeller mutations have yet been identified that are associated with normal rates of parasite clearance.^4^, ^5^, ^18^ Increasing evidence shows that away from areas of artemisinin resistance, mutations in the K13 propeller are not present at significant frequencies,^5^, ^21^, ^22^, ^23^, ^24^, ^25^, ^26^ and the total prevalence of the K13-propeller mutation is less than 5% in surveys from a range of transmission settings. Discovery of a molecular marker of artemisinin resistance before global spread provides a unique opportunity for surveillance to be done in near real-time to support containment and elimination strategies.^27^ WHO have incorporated results of K13 marker surveillance into a revised definition of artemisinin resistance.^1^

Myanmar stretches from the Bay of Bengal and Andaman Sea in the south to the Himalayas in the north, and therefore provides the only route for drug-resistant falciparum malaria to spread contiguously from southeast Asia to the Indian subcontinent,^28^ a path followed by resistance to chloroquine and probably pyrimethamine nearly half a century ago.^29^ Artemisinin resistance in *P falciparum* has been present at the Myanmar–Thailand border for several years,^6^, ^30^ and slow parasite clearance after artemisinin-based combination treatments has also been reported in southeastern Myanmar.^31^, ^32^ In 2011–12 in Shwe Kyin, Bago Region, roughly 15% of patients had delayed parasite clearance (a clearance half-life >5 h), and a quarter had mutations in the propeller region of the K13 protein.^5^ Before this study, the prevalence of artemisinin-resistant parasites in other parts of Myanmar had not been reported. Here, we present a detailed molecular survey of K13 based on *P falciparum* field isolates obtained from patients in Myanmar and neighbouring regions.

## Methods

### Study design and patients

We obtained samples for K13 genotyping from symptomatic patients presenting with fever to malaria treatment centres at 55 sites in ten administrative regions in Myanmar, and two cross-border areas in Thailand and Bangladesh, between August, 2013, and September, 2014. Patients had falciparum malaria confirmed by blood smear or rapid diagnostic test. For patients enrolled in prospective clinical studies, either whole blood samples or dried blood spots on filter paper were collected after fully informed consent was obtained. In a small subset of these patients, both the screening rapid diagnostic test and the whole blood sample were processed to check for concordance. For patients being managed with routine care, used rapid diagnostic tests were retained anonymously for later extraction of plasmodium DNA. Additionally, whole blood samples were collected between January and December, 2013, as part of ongoing epidemiological studies in villages and camps on either side of the Myanmar–Thailand border (in Tak province and Kayin State) and for hospital-based studies done between May and September, 2014, in Ramu, Bangladesh. This method led to unequal distributions of sample types and patient characteristics (clinical severity, age, sex, and pregnant state) across the various sites. However these factors were thought unlikely to significantly affect the overall prevalence of K13-marker mutations at each site. In accordance with therapeutic efficacy studies,^33^ we planned to obtain at least 50 sequences in each administrative division, although we anticipated that this might not be achieved for operational reasons and possible failure to obtain the necessary K13 sequence in a specific sample.

Ethics review and approval was obtained from the Faculty of Tropical Medicine, Mahidol University (Thailand); Department of Medical Research, Ministry of Health (Myanmar); The Defence Services Medical Ethics Committee (Myanmar); and Oxford Tropical Research Ethics Committee (UK). Samples from within Thailand and Bangladesh were obtained as part of prospective clinical studies covered by existing ethical approvals.

### Procedures

DNA was extracted from dried blood spots, completed rapid diagnostic test strips (both stored desiccated at room temperature), and frozen whole blood samples, by standard methods.^5^ Relevant primers derived from the K13 gene sequence were used to amplify the full kelch13 open-reading frame with use of a nested PCR protocol^5^ to describe the complete sequence from aminoacid 210 onwards (the conserved part of the protein). We regarded heterozygous results as mutations.

### Geospatial mapping

For the purpose of predictive geospatial mapping, we calculated the total proportion of samples in each location with a non-synonymous mutation after aminoacid position 440 of the K13 gene. These data, in addition to the total number of samples and geographical information system coordinates for each sampling site, were entered into a geostatistical model, producing a predictive map of estimated mutation prevalence on a 5 × 5 km grid in Myanmar. To reduce model uncertainty and achieve a robust estimate of the prevalence of the K13 mutation, we used two alternative geostatistical modelling approaches. The first approach involved use of a regression model implemented within a Bayesian framework, producing a posterior distribution of prevalences summarised by the median to create a single continuous surface as described previously (appendix).^34^ Second, we applied the well-established spatial statistical interpolation method of ordinary kriging.^35^ With both models we generated a corresponding uncertainty map to show the confidence associated with the predictions across the map domain. In the Bayesian model, this prediction error was represented by a corresponding SD surface, whereas in the kriging model it was shown by the kriging variance. With the kriging approach, we also used a variogram to describe the strength of spatial dependence shown in the data (appendix).

### Role of the funding source

The sponsor of the study had no role in study design, data collection, data analysis, data interpretation, or writing of the report. The corresponding author had full access to all the data in the study and had final responsibility for the decision to submit for publication.

## Results

**Figure 1:**
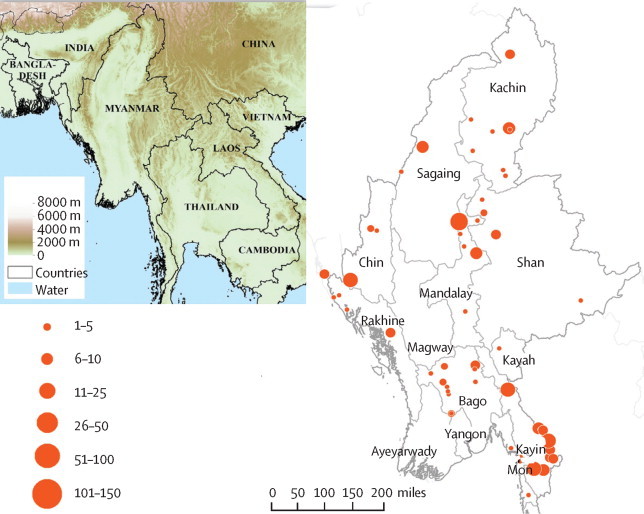
Location of sampling sites, sample sizes, and administrative states and regions of Myanmar, and a relief map of southeast Asia Red circles show numbers of patients in each region.

Figure 1 shows the location of sampling sites, sample sizes, and administrative states and regions of Myanmar. Of 2378 samples tested, 940 (40%) samples produced clear sequences covering aminoacids 210–726 of the K13 gene. The overall extraction–sequencing success rates were 97% for whole blood, 84% for dried blood spots, and 26% for rapid diagnostic tests. We did not screen samples with PCR before attempting to sequence K13. 11 of 12 samples sequenced from both rapid diagnostic tests and whole blood showed concordant sequences, with the remaining sample failing to produce a sequence from the diagnostic test. Only five mixed genotypes were apparent in sequencing reads.

**Figure 2:**
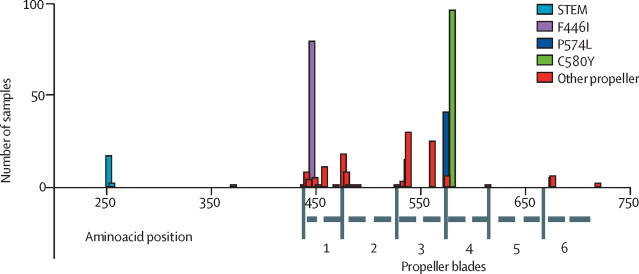
Primary aminoacid positions of K13 mutations identified in Myanmar and border regions

We identified 29 different mutations after aminoacid 210, of which 26 (90%) were after aminoacid 440 (appendix). 371 (39%) isolates had a propeller-domain mutation (table). Consistent with previous reports,^4^, ^5^ these mutations were concentrated within propeller blades 1–4 (figure 2). 17 isolates had an E252Q mutation in the so-called stem section of the K13 protein; one (2%) from Bago Region, 11 (4%) from Kayin State, and five (3%) from Tak Province. No sample had more than one mutation in these conserved domains of the protein.

**Table:** Number of samples per region and proportion with mutations in the propeller domain of K13

		**Total samples**	Samples with propeller mutation*	**Proportion (%)**
Myanmar				
	Bago	52	0	0 (0–6·9)
	Chin	62	2	3·2 (0·9–11)
	Kachin	70	26	37·1 (26·8–48·9)
	Kayah	2	2	100 (34·2–100)
	Kayin	288	137	47·6 (41·9–53·3)
	Mandalay	181	43	23·8 (18·1–30·5)
	Mon	8	3	37·5 (13·7–69·4)
	Rakhine	29	2	6·9 (1·9–22)
	Sagaing	46	21	45·7 (32·2–59·8)
	Shan	21	14	66·7 (45·4–82·8)
Bangladesh				
	Chittagong	25	0	0 (0–13·3)
Thailand				
	Tak	156	121	77·6 (70·4–83·4)
Total		940	371	39·5 (36·4–42·6)

Data in parentheses are 95% CI. We calculated confidence intervals with the Wilson Test (without continuity correction).

* After aminoacid 440.

**Figure 3:**
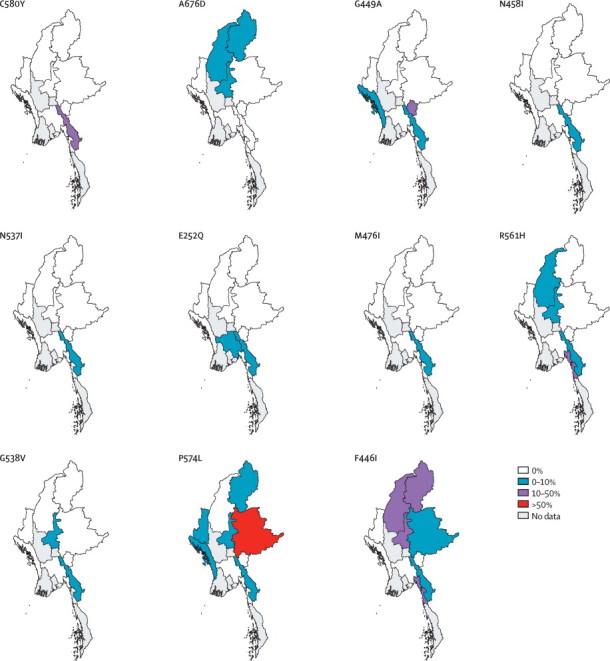
Local prevalence of individual K13 mutations by administrative state or region in Myanmar Only mutations found in at least nine isolates, or at least three states or regions, are shown.

Several mutations seemed to be concentrated in specific regions of Myanmar (figure 3). F446I was identified in 80 samples across six states or regions with several regions in Upper Myanmar showing prevalences in excess of 10%; 21 (47%) of 45 samples obtained in Homalin, Sagaing Region (25 km from the India border) had K13-propeller mutations (mostly the F446I mutation). The P574L mutation was also widely identified, being present in 41 samples across seven states or regions, whereas the A676D mutation was identified in three northern states or regions only (figure 3). The C580Y mutation, identified at high prevalence in western Cambodia,^4^ was confined to Kayin state, and was also present at high prevalence across the adjacent western part of Tak province in Thailand (figure 3). Notably, the M476I mutation, shown to develop after prolonged in-vitro artemisinin selection,^4^ was identified in 18 isolates.

Roughly two-thirds of the identified K13 mutations had been described previously in Myanmar or at the Myanmar–Thailand border,^5^, ^18^, ^36^ and a further subset of these had also been reported in Cambodia.^4^, ^5^ These previously identified mutations tended to have higher prevalences than those that were unique to Myanmar, none of which was identified in more than ten isolates (appendix). We identified three rare (three samples or less) synonymous mutations in the sequence after aminoacid 210 in the set of 759 samples from within Myanmar. Calculation of intraspecific evolutionary coefficients (with Jukes-Cantor correction) produced values of 0·0123 for dS and of 0·0136 for dN. Inference of selective forces from the resulting intraspecific dN to dS ratio of 1·10 is challenging, but the findings are compatible with strong positive selection.^37^

**Figure 4:**
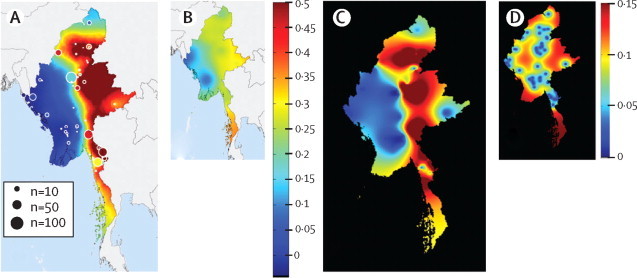
Geographical extent of predicted artemisinin resistance as determined by the prevalence of K13 propeller mutations (>440 aminoacids) visualised by approaches using a Bayesian model (A, with uncertainty shown in B) and kriging interpolation (C, with uncertainty shown in D) In the main prevalence maps, colour shows total prevalence of relevant K13 mutations (median in A and mean in C). In the uncertainty maps, orange and red areas show the greatest uncertainty in Shan State (in the east) and the southern peninsula. In A the colour of the circles is proportional to the recorded K13-mutation prevalence at a particular site and the radius of the circle is proportional to the sample size of the study.

We calculated the total prevalence of K13-propeller mutations for each administrative region (table) and site as the proportion of samples with any mutation after aminoacid 440. We entered these point metrics into two independent geospatial models to obtain continuous prevalence maps for Myanmar (figure 4). Both maps showed a large area of fairly high mutation prevalence (substantially more than 10%) extending from the southeast to the north of the country (figure 4). Much of Lower Myanmar, and Chin and Rakhine states in the west, had a very low prevalence of K13 mutations, a finding consistent with the absence of K13 mutant parasites in adjacent Bangladesh (figure 4).

## Discussion

Our findings provide strong evidence that artemisinin-resistant falciparum malaria extends across much of Upper Myanmar, including regions close to the Indian border in the northwest. By comparison, Lower Myanmar and the mountainous western states (Rakhine and Chin) currently have a relatively low prevalence of K13 mutations, and there is no evidence of spread into southeastern Bangladesh. There is also no evidence that artemisinin resistance has reached India; however, few data are available.^38^, ^39^ An independent study examining samples from 91 patients in Kayin state in east Myanmar and Chin state in the west likewise reported a range of K13-propeller mutations, with a higher prevalence in the eastern region and relatively few K13-mutant parasites at the western border with Bangladesh.^36^

Artemisinin resistance became established in western Cambodia more than a decade ago.^4^ Studies of population genetics have described *P falciparum* founder populations that share an underlying predisposed genetic background^40^ rising to fairly high levels; each population is linked to specific K13 mutations^41^ and has high levels of artemisinin resistance both in vivo and in vitro.^19^ Seven individual mutations seem to have arisen independently on more than one occasion in different locations; these mutations include C580Y, which is reaching fixation in various regions. In the present study, C580Y parasites were only located at the Myanmar–Thailand border, although this lineage seems to be separate to that in Cambodia.^18^ Few of the K13 mutants currently in Myanmar have been reported at high prevalence in Cambodia; furthermore, the Myanmar mutations seem to be clustered towards the first kelch domain (aminoacids 441–475) with one mutant in the conserved stem of the protein (E252Q) also reaching significant frequencies in southeast Myanmar. In the north of Myanmar, including sites very close to the Indian border, prevalence of the F446I mutation is high. Why there are different sets of mutations in different locations is not yet clear, but one possible explanation is that Myanmar is at an earlier stage of an evolutionary process than Cambodia. The prevalent mutations in Myanmar might provide reduced levels of artemisinin resistance (although the widespread P574L mutation seems to be associated with parasite clearance that is at least as slow as C580Y^5^, ^18^) or might bring fitness costs, so that they are outcompeted by fitter alternatives such as C580Y over time. Alternatively there might be distinctive selective forces resulting from different antimalarial use, host genetics, or mosquito biology that promote differential sets of mutations in the two regions. Further studies of parasite fitness and in-vitro drug sensitivity are likely to shed light on these questions.

This study shows that valuable real-time molecular epidemiological surveillance and monitoring can be done with used rapid diagnostic tests (and with dried blood spots) if there is a system to obtain these tests and send them to a reference laboratory. The two geospatial models described provide consistent and informative up-to-date knowledge of the extent of resistance to artemisinins in Myanmar and could be used to guide and prioritise interventions. Additional data are needed to reduce the uncertainty of the present estimates of resistance in specific locations, potentially guided by surveillance modelling methods.

The global spread of chloroquine resistance resulted in the loss of millions of lives in Africa and, clearly, Myanmar is an important part of the frontline in the battle to contain artemisinin resistance. These data emphasise the concern that artemisinin resistance could follow historical paths of the spread of antimalarial drug resistance from southeast Asia, via Myanmar, through India to Africa.^42^ Moreover, substantial increases in international travel and migration could promote direct spread of artemisinin resistance (so-called jumping).^43^ Local emergence of resistance parasite is an alternative scenario (so-called popping).

Myanmar has substantially more malaria than any other country in southeast Asia,^44^ so aside from the wide implications, artemisinin resistance could reverse recent downward trends in morbidity and mortality from malaria in the country. Knowledge of the level of K13-propeller mutations provides a snapshot of the extent of artemisinin resistance, but does not in itself provide direct information about the effectiveness of Myanmar's present first-line artemisinin-based combination treatment, artemether–lumefantrine. If, for example, the artemisinin resistance reported here results from early wide-scale availability of artemisinin monotherapy, the lumefantrine component could be sufficiently effective for the combination to retain high effectiveness in at least some regions of Myanmar. However, declining effectiveness of artemisinin-based combination treatments, representing a combination of artemisinin resistance and failing partner drug (mefloquine), is already a substantial problem on the eastern Myanmar border^14^ and, in view of the cross-resistance between mefloquine and lumefantrine, effectiveness in that region is probably poor. In other regions where K13-mutant parasites are prevalent, prediction of effectiveness is difficult, and therapeutic efficacy studies—the definitive method for identification of whether a combination is beginning to fail—are urgently needed, with a focus on the areas of emerging artemisinin resistance evident in this survey. Measurement of pfmdr1 copy number in these samples would also be useful in this respect.

Artemisinin resistance has not been contained. Present artemisinin-based combination treatments are failing in areas affected by artemisinin resistance and there is a real threat that the incidence of *P falciparum* will begin to rise again, thus confounding regional aspirations to eliminate malaria (panel). Even low numbers of recrudescences fuel the emergence and spread of resistance to the partner drug (which is exposed to a higher parasite burden because of reduced parasite killing by the artemisinin derivative), substantially shortening the lifetime of any artemisinin-based combination treatment.^45^ Switching to an alternative partner (such as piperaquine) successfully, but only temporarily, improved the effectiveness of artemisinin-based combination treatments in western Cambodia.^13^ These considerations suggest that malaria treatment needs to be revised. Use of regimens of more than 3 days' duration, or containing more than one partner drug, will become necessary across an expanding area of southeast Asia. The pace at which the geographical extent of artemisinin resistance is spreading is faster than the rate at which control and elimination measures are being developed and instituted, or new drugs being introduced. A vigorous international effort to contain this enormous threat is needed.

Panel **Research in context**

**Systematic review**

We searched PubMed from January, 2000, to January, 2015, with the terms “K13 or kelch” and “falciparum” and identified 18 papers. 13 papers, all of which were done between July, 2014, and January, 2015, and undertaken on the basis of the discovery of the K13 marker by Ariey and colleagues,^4^ reported molecular surveys of the K13 marker in field isolates. Mutations in the propeller region of the K13 protein (which consists of six kelch domains) cause artemisinin resistance in the ring stage of the parasite,^16^ although background parasite genetic traits are also important.^16^, ^40^ Findings from the Tracking Resistance to Artemisinin Collaboration study^5^ showed that artemisinin resistance was present in much of the lower Mekong region and that Myanmar spanned the divide between resistant and sensitive regions. The ability to establish the parasite's K13 sequence from blood samples stored at room temperature (as dried blood spots or discarded rapid diagnostic tests) provided an opportunity to assess the degree of artemisinin resistance in fairly remote areas.^27^

**Interpretation**

The long-term effectiveness of the present first-line treatment for falciparum malaria—artemisinin-based combination treatment—is dependent on the ring-stage killing action of artemisinins. Once this action is lost, artemisinins exert less antimalarial effect and resistance to the slower-acting partner drug is bound to develop over time. Our study shows that artemisinin resistance extends over more of southeast Asia than had previously been known, and is now present close to the border with India. This finding expands the area in which containment and elimination are needed to prevent the possibility of global spread of artemisinin resistance.
