# Supplementary material for: Spread of artemisinin-resistant Plasmodium falciparum in Myanmar: a cross-sectional survey of the K13 molecular marker
Source: Lancet Infect Dis. 2015 Apr;15(4):415–21. doi: 10.1016/S1473-3099(15)70032-0 (PMC4374103; doi:10.1016/S1473-3099(15)70032-0)
Supplement: Supplementary appendix [file mmc1.pdf]

THE LANCET Infectious Diseases

Supplementary webappendix

This webappendix formed part of the original submission and has been peer reviewed. We post it as supplied by the authors.

Supplement to: Tun KM, Imwong M, Lwin KM, et al. Spread of artemisinin-resistant *Plasmodium falciparum* in Myanmar: a cross-sectional survey of the K13 molecular marker. *Lancet Infect Dis* 2015; published online Feb 20. http://dx.doi.org/10.1016/S1473-3099(15)70032-0.

Supplementary Information

Model-based Method

A Bayesian geostatistical model of the proportion of individuals with any marker (>440 amino acids into the protein) was developed. The number of individuals in each study with a resistance mutation (N_i^+) was assumed to be binomially distributed, given the number of individuals in the study (N_i) and the probability $P(\underline{x}_i)$:

$$N_i^+ \left| N_i, P(\underline{x}_i) \sim Binomial(N_i, P(\underline{x}_i)) \right. .$$

The probability, $P(\underline{x})$, at an arbitrary location $\underline{x}$, was modelled as the inverse logit transformation of the sum of a random field, $f(\underline{x})$, and an unstructured random component, $\varepsilon(\underline{x})$:

$$P(\underline{x}) = \text{logit}^{-1}(f(\underline{x}) + \varepsilon(\underline{x})) .$$

The unstructured components, $\varepsilon(\underline{x})$, were assumed to be independent and identically distributed with zero mean and variance V while the random field, $f(\underline{x})$, was modelled as a stationary Gaussian process, with constant mean function $m(\underline{x}) = b_0$ and Matern covariance function, $C(\underline{x})$

$$f(\underline{x}) \left| b_0, s, j_x, g \sim GP(m(\underline{x}), C(\underline{x})) \right.$$

where b_0 is the mean parameter, j_x is the spatial scale parameter, s is the partial sill and g is the degree of differentiability parameter. The covariance between a study conducted at location $\underline{x}_i$ and a study performed at $\underline{x}_j$ was given by

$$C(\underline{x}_i, \underline{x}_j) = s^2 \frac{\text{D}x^g k_g(\text{D}x)}{2^{g-1}\text{G}(g)},$$

$$\text{D}x = \frac{2\sqrt{g} D_{GC}(\underline{x}_i, \underline{x}_j)}{j_x}$$

where $D_{GC}(\underline{x}_i, \underline{x}_j)$ is the great circle distance, Γ is the gamma function and κ_γ is the modified Bessel function of the second kind of order g. The following priors were imposed:

$$p(b_0) \,\text{μ}\, 1$$
$$f_x \sim \text{Exponential}(1)$$
$$V \sim \text{Exponential}(0.1)$$
$$s \sim \text{Exponential}(0.1)$$
$$g \sim \text{Uniform}(0, 3)$$

Using the Python module PyMC, the model was fitted with Markov chain Monte Carlo (MCMC) [1,2]. Predictive maps were generated on a 5 x 5 km grid from the MCMC samples. For each prediction location, prevalences were drawn and the distribution summarized with the median and standard deviation of this set.

1. Patil A, Huard D, Fonnesbeck C (2010) PyMC: Bayesian Stochastic Modelling in Python. Journal of Statistical Software 35: 1-81.

2. Patil A (2010) PyMC Gaussian process module Users guide.

Kriging Method

Let $z(x_k)$, k =1, 2 … n be the observed values of the variable z at locations $x_1, x_2, \ldots x_n$

We require to find coefficients λ_k for k = 1,2, … n, such that the estimate $\hat{z}_0$ of z_0 (=$z(x_0)$) at any point x_0

$$\hat{z}_0 = \sum_{k=1}^{n} \lambda_k z(x_k) \tag{1}$$

minimize the mean squared prediction error (referred to as 'the kriging variance')

$$\sigma_e{}^2 = E[(z_0 - \hat{z}_0)^2] \tag{2}$$

subject to the constraint (to ensure unbiased)

$$1 = \sum_{k=1}^{n} \lambda_k \tag{3}$$

2.1 Kriging coefficients w_k.

Denoting z_0 as a random variable and recalling that in ordinary kriging $E[z_0] = \mu$ where μ is an unknown but constant mean , we first note that

$$\sigma_e{}^2 = E[(z_0 - \hat{z}_0)^2] = Var\,(z_0 - \hat{z}_0) + E[(z_0 - \hat{z}_0)]^2$$

and because by unbiasedness $E[(z_0 - \hat{z}_0)]^2 = 0$,

$$= Var(z_0) + Var\,(\hat{z}_0) - 2Cov(\hat{z}_0, z_0) \tag{4}$$

Using the method of Lagrange multipliers we therefore require to minimize the Lagrangian

$$L \quad = Var\,(z_0 - \hat{z}_0) + 2\beta\left(\sum_{k=1}^{n} \lambda_k - 1\right)$$

$$= \sigma^2 + \sum_{k=1}^{n}\sum_{j=1}^{n} \lambda_k\, \lambda_j Cov\left(z(x_k), z(x_j)\right) - 2\left(\sum_{k=1}^{n} \lambda_k Cov(z(x_k), z_0)\right) + 2\beta\left(\sum_{k=1}^{n} \lambda_k - 1\right) \tag{5}$$

where σ^2 is the variance of z_0 and $Cov(x,y)$ is the covariance of x and y. This is achieved by taking partial derivatives of L with respect to λ_k for k= 1, 2, … n and the β and then setting each to zero, which yields a homogeneous system of n simultaneous equations which can be written in matrix form as

$$\begin{bmatrix} Cov(z(x_1),z(x_1)) & \cdots & Cov(z(x_1),z(x_n)) & 1 \\ \vdots & \ddots & \vdots & \vdots \\ Cov(z(x_n),z(x_1)) & \cdots & Cov(z(x_n),z(x_n)) & 1 \\ 1 & \cdots & 1 & 0 \end{bmatrix} \begin{bmatrix} \lambda_1 \\ \vdots \\ \lambda_n \\ \beta \end{bmatrix} = \begin{bmatrix} Cov(z(x_1),z_0) \\ \vdots \\ Cov(z(x_n),z_0) \\ 1 \end{bmatrix} \quad (5)$$

or more compactly as

$$\boldsymbol{C\lambda} = \boldsymbol{D} \quad (6)$$

The kriging coefficients λ_k and the Lagrange multiplier β are then found by multiplying the right hand side vector in equation (8) by the inverse matrix $\boldsymbol{C}^{-1}$

$$\boldsymbol{\lambda} = \boldsymbol{C}^{-1}\boldsymbol{D}$$

2.1 Kriging coefficients λ_k in terms of the variogram

The variogram can be written in terms of the covariance function as

$$\gamma_{ij} = \frac{1}{2} Var\left(z(x_i) - z(x_j)\right)$$

$$= 1/2(\sigma^2 + \sigma^2 - 2Cov(z(x_i) - z(x_j)))$$

$$= \sigma^2 - Cov(z(x_i) - z(x_j))$$

So we have that

$$Cov(z(x_i) - z(x_j)) = \sigma^2 - \gamma_{ij}$$

and the kriging equations (5) can be rewritten equivalently in terms of the variogram as

$$\begin{bmatrix} -\gamma_{11} & \cdots & -\gamma_{1n} & 1 \\ \vdots & \ddots & \vdots & \vdots \\ -\gamma_{n1} & \cdots & -\gamma_{nn} & 1 \\ 1 & \cdots & 1 & 0 \end{bmatrix} \begin{bmatrix} \lambda_1 \\ \vdots \\ \lambda_n \\ \beta \end{bmatrix} = \begin{bmatrix} -\gamma_{10} \\ \vdots \\ -\gamma_{n0} \\ 1 \end{bmatrix} \quad (7)$$

or more compactly

$$\boldsymbol{\Gamma\lambda} = \boldsymbol{\Gamma}_0 \quad (8)$$

Kriging coefficients λ_k and the Lagrange multiplier β are found multiplying the right hand side by the inverse matrix $\boldsymbol{\Gamma}^{-1}$

$$\boldsymbol{\lambda} = \boldsymbol{\Gamma}^{-1}\boldsymbol{\Gamma_0}$$

2.3 Kriging variance $\sigma_e{}^2$

Multiplying *i*th row of matrix **C** and **D** in equation (5) by λ_i for *i* =1 …*n* and forming their sum gives

$$\sum_{i=1}^{n}\lambda_i\sum_{j=1}^{n}\lambda_j Cov\left(z(x_i),z(x_j)\right)+\sum_{i=1}^{n}\lambda_i\,\beta=\sum_{i=1}^{n}\lambda_i Cov(z(x_i),z_0)$$

From equation (4) the kriging variance is then given by

$$\sigma_e{}^2=\sigma^2+\sum_{k=1}^{n}\sum_{j=1}^{n}\lambda_k\,\lambda_j Cov\left(z(x_k),z(x_j)\right)-\;2\left(\sum_{k=1}^{n}\lambda_k Cov(z(x_k)\,,z_0)\right)$$

$$=\sigma^2+\left(\sum_{i=1}^{n}\lambda_i Cov(z(x_i),z_0)-\beta\right)$$

$$-\;2\left(\sum_{k=1}^{n}\lambda_k Cov(z(x_k)\,,z_0)\right)$$

$$=\sigma^2\text{--}\;\boldsymbol{\lambda}^{\boldsymbol{T}}\mathbf{D} \qquad (9)$$

where $\boldsymbol{\lambda}^{T}$ denotes the transpose of vector **λ** from equation (6)**.**

2.4 Kriging variance $\sigma_e{}^2$in terms of the variogram

The kriging variance $\sigma_e{}^2$ can similarly be expressed in terms of the variogram by replacing covariance terms in vector **D** of equation (8)

$$\boldsymbol{D}=\begin{bmatrix}Cov(z(x_1),z_0)\\ \vdots\\ Cov(z(x_n),z_0)\\ 1\end{bmatrix}=\begin{bmatrix}\sigma^2-\gamma_{10}\\ \vdots\\ \sigma^2-\gamma_{n0}\\ 1\end{bmatrix}$$

so that from equation (9)

$$\sigma_e{}^2=\sigma^2-\;\boldsymbol{\lambda}^{\boldsymbol{T}}\boldsymbol{D}$$

$$=\sigma^2-[\lambda_1\quad\ldots\quad\lambda_n\quad\beta\,]\begin{bmatrix}\sigma^2-\gamma_{10}\\ \vdots\\ \sigma^2-\gamma_{n0}\\ 1\end{bmatrix}$$

$$=\sigma^2-\sigma^2\sum_{i=1}^{n}\lambda_i-\sum_{i=1}^{n}\lambda_i\gamma_{i0}+\beta$$

$$=\sum_{i=1}^{n}\lambda_i\gamma_{i0}-\beta$$

Supplementary Table

List of mutations found in 940 samples

Mutation	Number of samples with mutation	Number of States/Regions where mutation was found	Notes and references
**Unique to this survey**			
N371I	1	1	
P443S	4	2	
N458I	9	1	
S485N	1	1	
N490T	1	1	
P527H	1	1	
G533A	3	2	
A675V	5	2	
A676D	6	3	
**Previously reported in Myanmar or Myanmar-Thailand border** [5, 18, 36]			
E252Q	17	3	
K438N	1	1	
P441L	8	2	
F446I	80	6	
G449A	5	4	Also reported in Cambodia[4]
D452E	1	1	
N458Y	2	1	Also reported in Cambodia[4, 5]
C469F	1	1	
M476I	18	2	
K479I	8	1	
A481V	1	1	Also reported in Cambodia[4]
N537I	15	2	Also reported in Cambodia[4]
G538V	30	3	
R561H	25	5	Also reported in Cambodia[4]
P574L	41	7	Also reported in Cambodia[4]
R575K	6	2	
C580Y	97	2	Also reported in Cambodia[4, 5]
F614L	1	1	
**Previously reported in Cambodia only**[5]			
H719N	2	1	
**Previously reported in Africa only**[5]			
R255K	2	2	

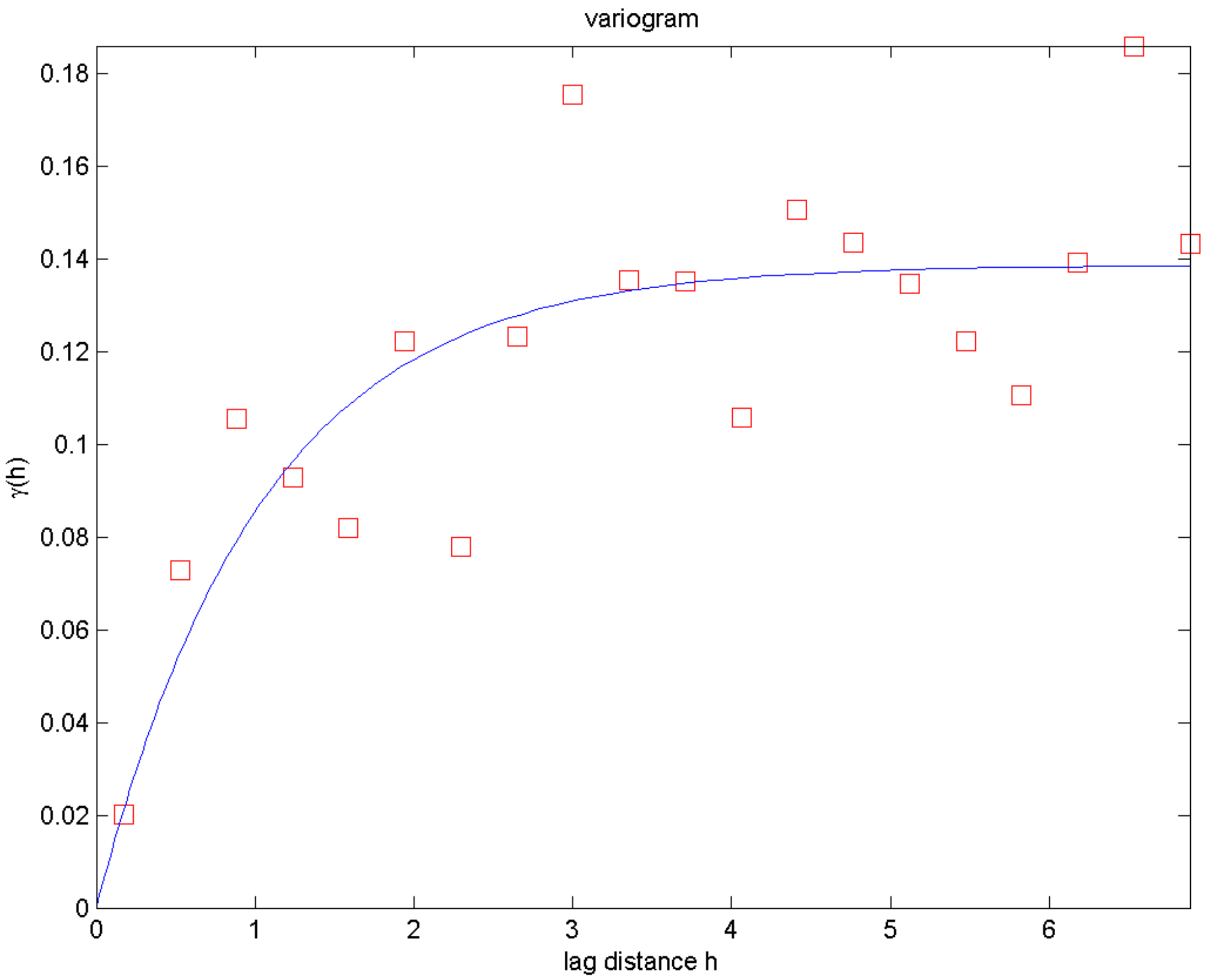

Supplementary Figure 1

Variogram produced by the kriging approach. An empirical variogram was constructed as a scatter plot (squares) of semivariance *γ(h)* versus lag distance *h* to describe spatial dependence in the data collected. The plot describes how variation in estimated K13 mutation prevalence observed between different sample sites is dependent on (Euclidean) separation distance between those sites. The plot was constructed by binning all pairwise site distances into a default 20 bins, each at a fixed increment apart. Spatial dependence can be seen to decrease as the distance between the sample data sites is increased. Such a plot will typically achieve a plateau (referred to as a 'sill') after a certain separation distance (referred to as the 'range') exhibited by a ‘flattening of the curve’ as is illustrated in the plot after a lag distance of approximately 4 separation units (20 km).
